# Analysis and Validation of Varied Simulation Parameters in the Context of Thermoplastic Foams and Special Injection Molding Processes

**DOI:** 10.3390/polym15092119

**Published:** 2023-04-28

**Authors:** Dimitri Oikonomou, Hans-Peter Heim

**Affiliations:** Institute of Material Engineering, Polymer Engineering, University of Kassel, 34125 Kassel, Germany; heim@uni-kassel.de

**Keywords:** simulation, Moldex 3D, thermoplastic foam, cell morphology, polycarbonate, computer-aided engineering, injection molding processes

## Abstract

The simulation solutions of different plastic injection molding processes are as multifaceted as the field of injection molding itself. In this study, the simulation of a special injection molding process, which generates partially foamed integral components, was parameterized and performed. This partial and physical foaming is realized by a defined volume expansion of the mold cavity. Using the injection molding simulation software Moldex 3D, this so-called Pull and Foam process was digitally reconstructed and simulated. Since the Pull and Foam process is a special injection molding technique for producing foamed components, the validity of the simulation results was analyzed and evaluated. With the use of Moldex 3D, varied settings such as different bubble growth models and mesh topologies were set, parameterized, and then analyzed, to provide differentiated numerical calculation solutions. Actual manufactured components represent the experimental part of this study and are produced for reference. Different evaluation methods were used to quantify morphological quantities such as porosities, local densities, and cell distributions. These methods are based on two-dimensional and three-dimensional imaging techniques such as optical microscopy and X-ray microtomography (µ-CT). Thus, this structural characterization of the manufactured samples serves as the validation basis for the calculated results of the simulations. According to the illustrated results, the adequate selection of bubble growth models and especially mesh topologies must be considered for valid simulation of specific core-back techniques, such as the Pull and Foam injection molding process.

## 1. Introduction

A variety of methods of physical foam injection molding (FIM) techniques of plastic components, allow the generation of microcellular integral components, which can offer many advantages. Among the benefits are a density reduction of up to 60% and a local increase in stiffness [1,2]. Furthermore, advantages such as a reduced degree of warpage can be obtained [3]. In other articles, a first approach has already been made to describe the relationship between process parameters, morphologies, and mechanical properties [4]. The use of multiple linear regression models was carried out, with which the influence of variables such as the cell distance, homogeneity, sphericity, and cell density on the flexural strength could be described [4]. In further publications, Heim and Güzel also provide insights into the effects of process parameters on structural and mechanical parameters on the basis of experimental studies [5]. In addition to descriptive data-based modelling, numerical solutions can be applied in the form of simulations. Since the research and development regarding the realisation of various intended structural and mechanical properties requires a great number of experiments, the simulation of foam injection molding processes offers itself as an alternative approach to gain new insights. These simulations provide the advantage of being able to perform a computer-aided engineering without the need for an experimental part. However, a proper setting and parameterization of simulations is required to make valid predictions. In this article, the filling simulation of a special physical foam injection molding process called Pull and Foam is parameterized and evaluated using various simulation settings.

**Figure 1:**
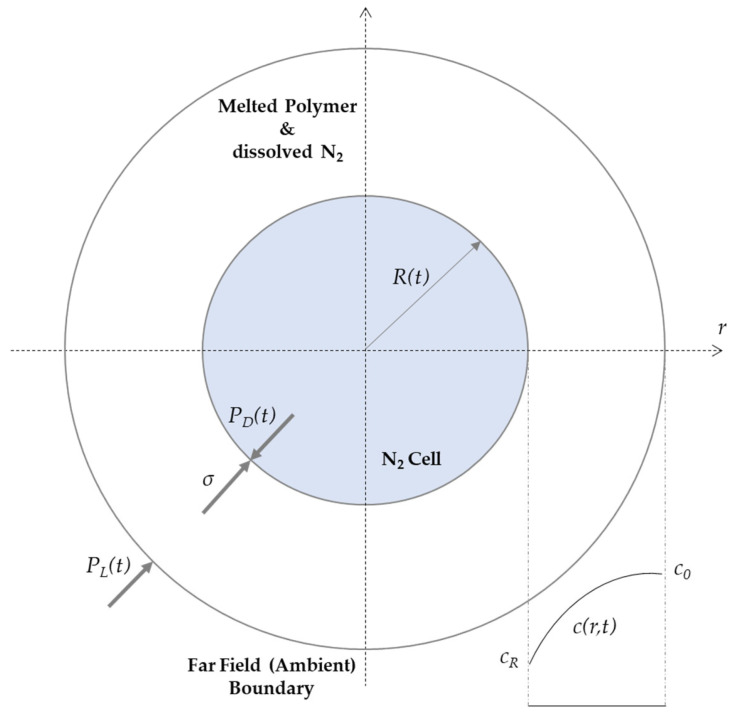
Illustration of relevant variables in the context of nucleation and growth of a single cell.

In this study, the simulation software Moldex 3D (CoreTech System Co., Ltd., Zhubei, Taiwan) is used, which includes the bubble growth models Han and Yoo (standard version), Han and Yoo—Modified, Payvar, Shafi, as well as Rosner as equations for cell nucleation and cell growth. These model equations are partially elaborated in the following sections and are also based on the findings and explanations in [6,7,8,9,10,11]. These past studies and experiments have already investigated how various bubble growth models perform in different theoretical and experimental scenarios. In particular, the nucleation of a cell in a supersaturated solution is described, which takes place as soon as a pressure drop is initiated and a thermodynamic instability is induced [12]. At the same time, the growth of the nucleated cells takes place, which grows until an equilibrium is reached [13,14]. To simulate the dynamics of cell dimensions, the models mentioned above are based on the nucleation and growth of single cells, respectively bubbles. Figure 1 shows the most important parameters that are represented in the equations used and is based on [13,14,15]. Here, *R* represents the cell diameter, *P_D_* the bubble pressure, while *P_L_* can be equated with the saturation pressure at *t* = 0 s. The concentration variable *c*(*r, t*) characterizes the variable N_2_ concentration as a function of radial position *r* and time *t*. The variable *σ* furthermore represents the surface tension of the bubble.

In summary, it can be expressed regarding cell growth, that in general the Shafi model has the slowest growth rate of bubbles. Han and Yoo, on the other hand, is shown to be faster. The Payvar model shows the most rapid growth rate of bubbles in theory. The Han and Yoo—Modified and Rosner models, on the other hand, were designed for high-pressure systems in foam injection molding processes [13,16].

In this study, two different types of mesh configurations, respectively methods, are investigated. First, the simulated foamed sample, respectively the expansion zone, which in reality is realized by a core back technique, is meshed with a structured and uniform mesh of pure triangular prismatic (pentahedral) elements. These polyhedral elements, such as triangular prismatic and hexahedral elements, exhibit higher mesh quality compared to simpler elements (such as tetrahedral elements), as they are less sensitive to stretching. Thus, they are also less sensitive to increasing skewness and are more likely to show an ideal aspect ratio of 1. A poor aspect ratio results in longer calculation times and in a reduction of the simulation accuracy of the solver [17]. In addition, high aspect ratios cause significant interpolation errors. With the same number of elements, polyhedral elements lead to a higher calculation accuracy than tetrahedrons [18]. Therefore, an objective of this study was to investigate the accuracy of the simulation results with pure uniform triangular prism meshes (pentahedral elements). Tetrahedral elements are highly flexible and the simplest element types and are particularly suitable for automated meshing of complex geometries. Boundary Layer Mesh (BLM) configurations are partially structured hybrid meshes consisting of triangular prismatic/hexahedral elements along the boundary walls and tetrahedral elements at the center region. The boundary regions are meshed with layers of triangular prismatic or hexahedral elements (prisms). The center of the geometries, on the other hand, is meshed with unstructured tetrahedrons, which serve as flexible connecting elements [19]. BLM configurations are used where boundary regions require high computational accuracy and center regions can operate with lower computational accuracy. This study therefore used the BLM configuration as the second meshing method and investigated whether a purely complete meshing with triangular prisms (high, uniform resolution and high element quality in all regions) or an efficient meshing with a BLM configuration (above-average resolution in boundary regions and lower resolution and element quality at the center) would prove to be favourable in the context of the Pull and Foam process simulation.

**Figure 2:**
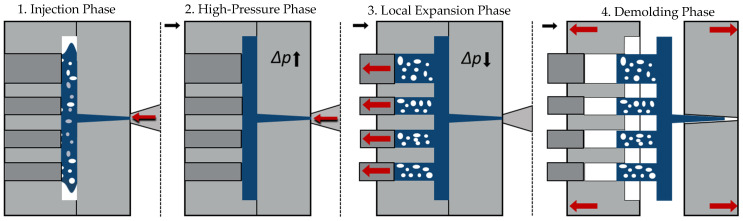
Schematic representation of a cycle of the Pull and Foam process.

**Figure 3:**
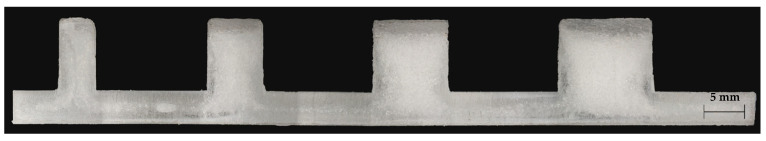
Cross-section of a foamed sample with a rib height of 6.5 mm each.

The Pull and Foam process is based on the development and research of Heim and Tromm [3,20] and describes the physical foaming of thermoplastic polymers and is based on a precision mold opening technology (PMO) [21]. In contrast to a classical low-pressure PMO, the Pull and Foam process does not expand the entire cavity, but rather the individual locations using a core back technique. After filling of the complete cavity, individual local sub-cavities (ribs) are expanded after a high-pressure packing time of up to 4 s (shown in Figure 2). Due to the local pressure drop as well as the applied delay and packing times distinct compact skin layers (with a thickness of approximately 0.5 mm) are the result, delineating the individual expanded ribs, which contain the foam structure in the core (shown in Figure 3). Therefore, the first phase can be described as the Injection Phase, in which the plasticized and homogenized melt is injected into the cavity. Due to the initial pressure drop, the previously observed mechanisms, such as sorption and diffusion, are revised. Consequently, an initial nucleation and growth of bubbles occur in the first phase [3]. In the second phase, which can be described as the High-Pressure Phase, a high-pressure level of up to 100 MPa is established by the packing pressure. Diffusion and sorption mechanisms induce a re-homogenization of the polymer melt, which can be observed by a shrinkage of the bubbles that were previously formed. A uniform and finer foam structure is therefore the result of this dissolution of the cell structure [22]. In the third phase called Local Expansion Phase, a pressure decrease in the supercritical fluid (SCF) is initiated by expanding the local cavities (ribs) using the core-back technique. Consequently, in this phase, a re-nucleation and growth of the bubbles is observed, which ultimately constitute the foam structure [20]. According to Park et al. [14], in the first instances of cell growth, there is a significant expansion of the cell, which results in a decrease in concentration in the cell, which in turn drives further progression of diffusion. Phenomena such as cell rupture and cell coalescence additionally occur in a simultaneous process. During the final phase, which is called the Demolding Phase, the foamed component is cooled and demolded. Furthermore, the nucleation and cell growth is consequently completed, since equilibrium has been achieved and no more energy is available to be dissipated [14]. The individual foamed ribs can thus be mechanically processed into a wide variety of specimens.

When it comes to Moldex 3D simulations of physical foam injection molding processes respectively MuCell^®^ processes, the effect of different numeric models and simulation parameters have already been studied to some extent. For example, it has been demonstrated that an increasing blowing agent content significantly reduces the cell diameter [23]. These findings were further confirmed by [24,25] on an experimental level. In addition, there have been dedicated numerical studies of mere bubble growth models describing the nucleation and growth of cells. Among other aspects, Venerus [26] investigated as well how the effect of the blowing agent concentration affects the dynamics of cell growth. The influence of the melt temperature was also investigated for the simulation with Moldex 3D. As a result, Ding, Vyas et. al. [23] reported that the effect of this factor on the cell structure remains relatively minor. An increase in the melt temperature therefore increases the cell diameter only insignificantly. In [27], the focus of the research was the determination of nucleation parameters, such as the correction factor for nucleation energy *F* and nucleation factor rate *f_0_*. Thus, for the system PP/nano-CaCO3 these factors can be calculated by means of nonlinear regression on experimental cell size and density distribution. Further publications were dedicated to the comparison of microscopic examinations and MuCell^®^ simulations using Moldex 3D. Among other findings, it was shown that the cell distribution differed significantly. Hence, this particular study pointed out that in the simulation of this process, larger cells were calculated in the areas of the cavity boundary (end of flow) and at the boundary regions of the sample [28]. Ding et al. [29] critically investigated the simulation accuracy of the Moldex 3D software and showed that an optimization of the mathematical models is required for many cases. Uniform cell nucleation, as calculated by Moldex 3D, led to results that still need to be optimized with regard to the foam injection molding process under investigation. In addition, no individual cell shapes were calculated, which are, however, highly relevant for mechanical properties. By comparison with other software solutions such as Moldflow (Autodesk, San Rafael, CA, USA) and Cadmould (Simcon Kunststofftechnische Software GmbH, Würselen, Germany), Moldex 3D proves to be the most accurate simulation software [30,31,32]. In [33], the accuracy of the simulation as a function of the weight reduction was investigated. Thus, [33] concluded that the Han and Yoo model achieves good simulation accuracy for the cell diameter and cell density especially in the context of a weight reduction of 5 wt%. There is, however, a need for a study focusing on the variation of typical simulation parameters in the context of high-pressure foam injection molding processes, which allow local expansion of different cavities.

Therefore, in this article, different Moldex3D simulation settings and parameters such as mesh type configurations and bubble growth models were varied to evaluate simulation performance and accuracy in the context of the specific foam injection molding technique called Pull and Foam. Thus, this study analysed and validated different bubble growth models and mesh configurations for this specialized process. As previously described, this process is based on a precision opening injection mold with local core pulling in the cavity (core back technology). To validate the simulated quantities, such as the cell size and component density, microscopy images as well as X-ray microtomography (µCT) images were used in the experimental part of this study.

## 2. Materials and Methods

The produced samples in this paper feature a dimension of 120 mm × 80 mm × 3 mm (initial thickness) and exhibit a total of four expanded ribs, which have varying widths of 4, 6, 8, and 10 mm respectively. The variable height of these expanding ribs ranges from 0 mm to 12 mm. Figure 3 shows the cross-section of a foamed sample. In this case, the expansion respectively the rib height amounts to 6.5 mm.

### 2.1. Machine and Foam Injection Molding Parameters

The Pull and Foam Process is conducted with a MuCell^®^ enhanced Arburg Allrounder 470S (Arburg GmbH + Co KG, Loßburg, Germany) injection molding machine. The machine equipment also includes a MuCell^®^-screw (25 mm), a shut-off nozzle, and a hot-runner system. The maximum clamping force amounts to 1100 kN. In addition, the maximum screw stroke extends to a maximum of 120 mm while the maximum stroke volume equals 59 cm^3^. Furthermore, the maximum screw torque amounts to 210 Nm. The heating power on the machine used totals 9.4 kW. Using the MuCell^®^ technology, a physical blowing agent (N_2_) is fed into the molten polymer.

As already stated, the rib height in this study equals 6.5 mm. The nitrogen respectively blowing agent content amounted to 0.4 wt% (mass fraction) and the polymer used was a pure polycarbonate (see Section 2.2). The injection flow rate was furthermore set at 50 cm^3^/s. The temperature profiles were configured in such a way that the melt temperature amounted to 290 °C and the mold temperature to 80 °C. The hot runner which was employed also featured a temperature of 290 °C. Here, it should be emphasized further that the packing phase (respectively High-Pressure Phase as shown in Figure 2) persists from the complete filling of the cavity and up to the local volume expansion of the individual cavities (ribs). In this case, the level of packing pressure amounted to 60 MPa, which was applied for 2 s. In addition, the core back speed was specified with 20 mm/s. Prior to demolding the sample, the final cooling time amounted to 45 s.

### 2.2. Materials

The used material was a pure polycarbonate, which is marketed by Covestro AG (Leverkusen, Germany) under the brand Makrolon^®^ 2405. Table 1 illustrates the most important processing properties of the material.

### 2.3. Three Dimensional X-ray Microtomography (µCT)

For the following measurements, the measuring device with the designation Zeiss XRadia520 Versa microscope (Zeiss, Oberkochen, Germany) was utilized.

**Figure 4:**
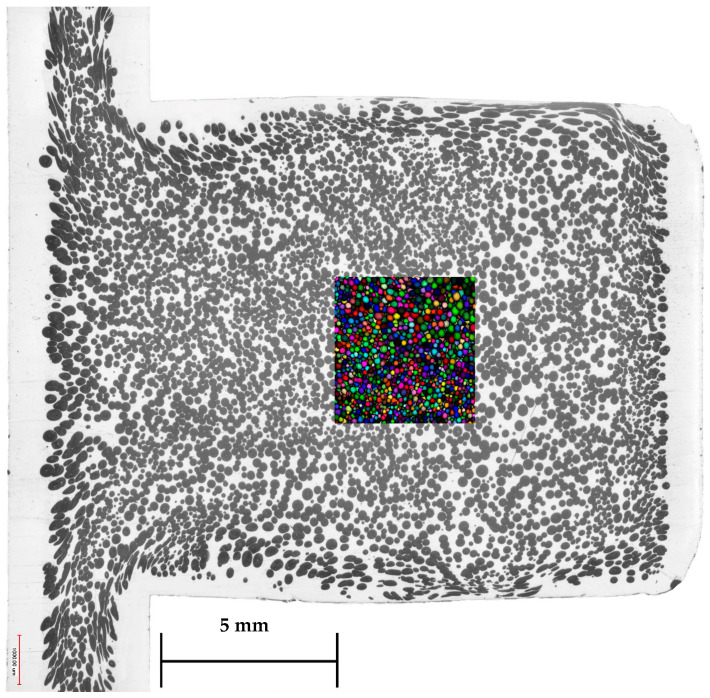
Measuring volume (colored area) of the X-ray microtomography images with the dimensions of 3000 µm × 3000 µm × 3800 µm.

This three-dimensional imaging technique measures a volume of 3000 µm × 3000 µm × 3800 µm (as shown in Figure 4) with a resolution of 1.995 µm. For the evaluation, the software Avizo (FEI, Hillsboro, OR, USA) was used, with which the quantities, such as length and width of the bubbles, as well as a metric regarding the sphericity can be measured and displayed. An ideal spherical shape would thus be declared with the value of 1.

### 2.4. Digital Light Microscopy

**Figure 5:**
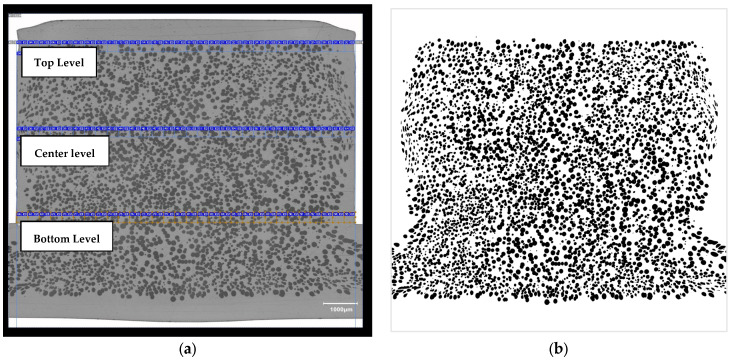
(**a**) Microscopy image (cross-section) of a foamed rib with all three levels with 30 data points each for local density evaluation; (**b**) Modified high-contrast image, for further use in Photoshop and Avizo.

The digital light microscope used holds the brand name Keyence VHX (Keyence, Osaka, Japan). For the microscope images, the foamed sample was analysed in cross-section (shown in Figure 5). To prepare the produced samples for the microscopy images, a section was dissected out, ground, and embedded in resin. For the structural characterization of the cells, the software Avizo and Photoshop (Adobe Inc., San Jose, CA, USA) were used. Figure 5a shows an example of the analysis of the local component density using Photoshop on the microscopy images. Here the trend of the component densities on three levels (top level, center level, as well as bottom level) were determined. Thus, it was possible to determine local densities for all levels, each including 30 data points.

This technique, explained in the next paragraphs, is based on the works of [34,35] and was used for the following analysis. A density distribution over the cross-section of the sample can be displayed with images from reflected digital light microscopy. An overview image of the sample was converted into a gray scale image (black/white) for this purpose (as shown in Figure 5b). Thus, in each case, the bright areas (white) represent the matrix and the black areas represent the cells. Using the image processing software Photoshop from Adobe, the overview image can be divided into any number of segments with the tool “Slices”. For the analysis of the samples, 30 slices were used in each case. Since a value of the local density is determined for each individual area of a slice, a density distribution can be generated over the cross-section of the sample. From the number and size of the slices, the number of measurement points and the size of the measurement areas of the local density over the cross-section are thus obtained. In the further process, an average gray scale value is yielded for each slice using the software Photoshop. This is composed of the maximal gray scale value of 255 (white) and the minimal gray scale value of 0 (black). Using the density of the corresponding polymer, the data sheet for Makrolon^®^ 2405 displays, that polycarbonate has a density of 1.20 g/cm^3^. Finally, the maximal gray scale value and a conversion factor *CF* can be determined.

The conversion factor (sown in Equation (1)) is needed to determine the value of the existing local density with the average gray scale value.

(1) $$\mathrm{Conversion}\;\mathrm{Factor}\;\mathrm{CF}=\frac{\mathrm{Density}\;\mathrm{of}\;\mathrm{Polymer}}{\mathrm{Maximum}\;\mathrm{Gray}\;\mathrm{Scale}\;\mathrm{Value}}$$

The final local density to be yielded for each data point is calculated using the following Equation (2):

(2) Local Density=CF×Mean Gray Scale Value

### 2.5. Simulation Settings of Moldex 3D

**Figure 6:**
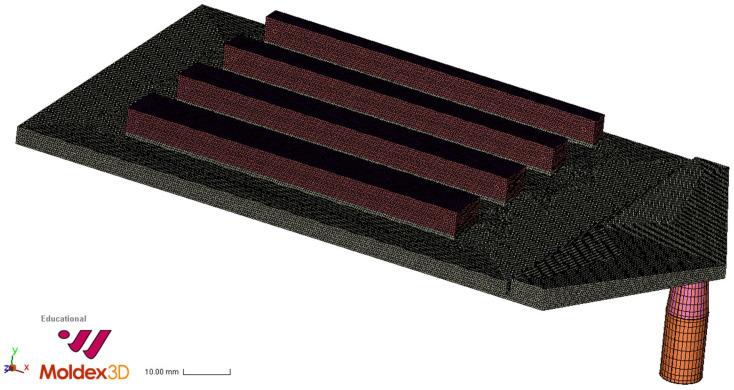
Moldex 3D interface with BLM meshed Pull and Foam sample (mesh size of 0.5 mm).

The simulation performed with the Moldex 3D 2022 software does not include any variations of typical injection molding process parameters, such as temperature and pressure profiles as already described, merely the five bubble growth models (Han and Yoo, Han and Yoo—Modified, Payvar, Shafi, and Rosner). The two mesh type configurations (uniform triangular prism mesh and hybrid BLM) were varied in an experimental design in order to investigate the effect of these parameters. Regardless of the mesh type, a mesh size or node distance of 0.5 mm was defined. In the case of a BLM meshing, the expansion zone (shown as red volumes in Figure 6) was parameterized with a boundary layer count of 10 and a boundary layer offset ratio of 3. In the second mesh configuration (uniform prism mesh), the expansion zone was meshed with triangular prismatic elements. This mesh was generated with a maximum layer count of 50 in the direction of the expansion (*Y* axis in Figure 6). It should be noted that Moldex 3D limited the layer count to 50.

The advanced foam settings, including nucleation parameters such as nucleation factor *f*_0_ and correction factor *F*, were kept at the default settings. The calculation time ranged from 18–20 h depending on the mesh types and the bubble growth models. The used computer system is based on a ×64 type and features an Intel^®^ Xeon^®^ Gold Processor 5220R (2.20 GHz, 24 cores). The RAM capacity amounts to 192 GB.

In general the growth of the individual cells is characterized by the coupled calculation of momentum and mass equations [36]. Representatively, the bubble growth models according to Han and Yoo (Equation (3)) and Han and Yoo—Modified (Equation (4)) are presented here, which describe the equation for mass transfer at the boundary of the cell as follows [6,23]:

(3) $$\frac{dP_{D}}{dT}=\frac{1}{R2} [\frac{6D\left(R_{g}T\right)\left(\bar{c}-c_{R}\right)}{-1+{\left\{1+\frac{2/R^{3}}{R_{g}T}\;\left(\frac{P_{D}R^{3}-P_{D0}R_{0}{}^{3}}{\bar{c}-c_{R}}\right)\right\}}^{0.5}}]-\frac{3P_{D}}{R}\frac{dR}{dt}$$

Here, *R_g_* describes the gas constant, $c_{R}$ the concentration of blowing agent gas at the bubble surface, and $\bar{c}$ the average concentration of dissolved blowing agent gas. *D* represents the diffusion coefficient, *T* the temperature, and *R* the cell radius. $P_{D}$ describes the bubble pressure and $P_{D0}$ the saturation pressure.

(4) $$\frac{dP_{D}}{dT}=\frac{1}{R^{2}} [\frac{6D\left(R_{g}T\right)\left(\bar{c}-c_{R}\right)}{-1+{\left\{1+\frac{2/R^{3}}{R_{g}T}\;\left(\frac{P_{D}R^{3}-P_{D0}R_{0}{}^{3}}{sgn\left(\bar{c}-c_{R}\right)(\bar{c}-c_{R})}\right)\right\}}^{0.5}}]-\frac{3P_{D}}{R}\frac{dR}{dt}$$

The other models can be examined in [7,8,9] as already mentioned. For instance, the Shafi model was parameterized with simultaneous nucleation as well as cell growth [37].

## 3. Results and Discussion

**Figure 7:**
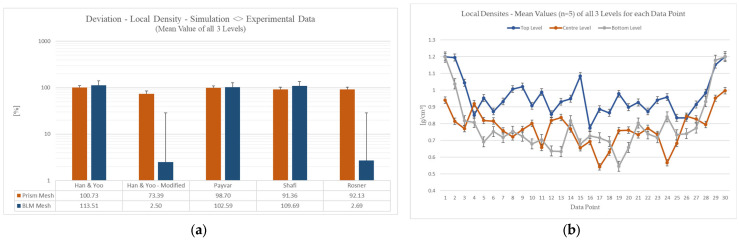
(**a**) Illustration of the deviation of the local density between experimental and simulation data depending on the bubble growth model and the mesh type; (**b**) Mean values for the local densities for all levels for each data point.

For a first illustration, Figure 7a shows the average percentage deviation, respectively the simulation error of the local density. Here, the respective trend of the density values of all three levels (top, center, and bottom level) and thus for each data point was averaged (shown in Figure 5a) and put into relation with the simulation data. This implies that each level, consisting of 30 data points of local densities, was first averaged. The resulting three mean density values for the top, center, and bottom level were then averaged once more, in order to have one global metric for the local density for one parameter combination like for example the Han and Yoo and BLM configuration. This aggregation method was analogously performed for the simulation data points, which also generated a final local density value. In Figure 7b it can be seen how the foam structure displays a typical integral profile. Hence, the local density at the edge of the compact skin layer amounts to 1.2 g/cm^3^. In the direction towards the center, a gradient can be determined which corresponds to decreasing local density. At the edge of the compact skin layer, it can be seen that the chart for the center level (orange graph) does not correspond to the density of the compact poly carbonate of 1.2 g/cm^3^ (shown in Figure 7b). This can be explained by the fact that during the Pull and Foam process the core back technique produces a tear-through of this compact layer at the center level. This phenomenon has also been observed in similar experiments [1]. As previously described, the percentage deviation, which is illustrated in Figure 7a, represents the averaged percentage deviation or error of the simulation data compared to the experimental data. The percentage deviation is displayed with absolute values and in logarithmic format for illustrative purposes. The comparison to the experimental data in Figure 7 is furthermore based on digital light microscopy images, as described in Section 2.4. As explained previously, all simulative and experimental conditions not listed here (process parameters, simulation parameters, etc.) were kept constant, so that the effects of these factors were factored out.

Initially, it can be concluded that—regardless of the mesh type—the bubble growth models Han and Yoo, Payvar, as well as Shafi generate an average deviation of about 100%. Considering the combination of a BLM configuration and the Han and Yoo—Modified model, it can be postulated that the local density can be simulated and thus predicted with an average percental error of 2.5% for the special injection molding process Pull and Foam. In addition, the Rosner model with a BLM configuration simultaneously simulates a minor error of 2.69%. The relatively wide variance can be explained by the fact that in the experimental part the effect of coalescence can occur, which can lead to a merging of individual cells. Similar findings were made in [33].

**Figure 8:**
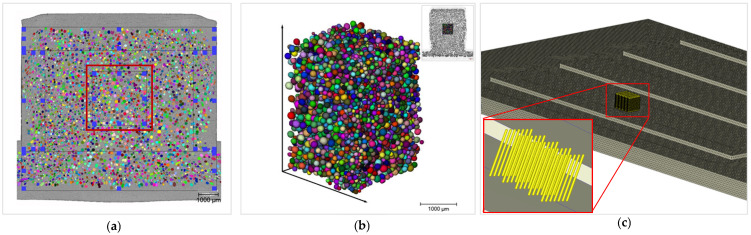
Measuring area: (**a**) Digital light microscopy (cross-section of the sample) with center area (highlighted red). (**b**) Measuring volume: X-Ray microtomography. (**c**) Replicated measurement volume in the injection molding simulation with Moldex 3D (2000 sensor nodes respectively data points).

In order to perform a more differentiated study to analyze the effects of the bubble growth models as well as the mesh types, structural parameters such as local density and cell size are henceforward evaluated for the center volume of the sample (Figure 8a—highlighted red). The use of X-ray microtomography also allows the generation of three-dimensional data, which corresponds to a more valid and more comprehensive database to compare simulation data with experimental morphological data (Figure 8b). The following data were generated by replicating the represented measurement volume of the X-ray microtomography and the associated measurement points using sensor nodes in the simulation (shown in Figure 8c). The geometric dimensions of the measurement volume of 34.2 µm^3^ were kept identical to the X-ray microtomography measurements. Consequently, the measurement volume in the simulation contains a number of 2000 sensor nodes. To subsequently compare consolidated morphological quantities, such as cell diameter and local density, the values of all data points were averaged. Since in the simulation cell sizes have an ideal sphericity of 1, an adapted evaluation of the cell sizes of the X-ray microtomography images is required. Following an average sphericity of 0.986 (standard deviation of 0.00557), an ideal sphere can be presupposed for the real microcellular bubbles (cells). Based on the measured volumes of the respective cells, the diameter was subsequently calculated in order to make the following comparisons.

**Figure 9:**
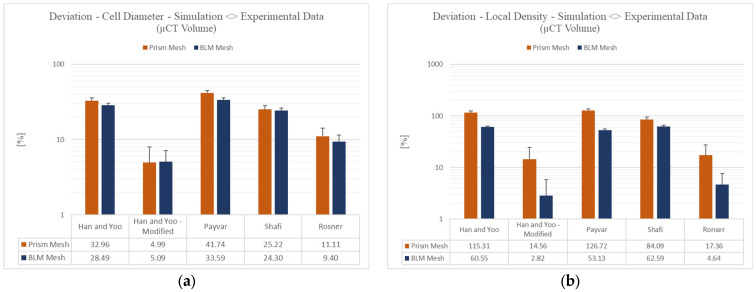
(**a**) Simulated deviation (error) of the mean cell diameter. (**b**) Simulated deviation (error) of the average local density.

Looking at the results in Figure 9, it can be verified from the upper first finding, that the simulation with the bubble growth models by Han and Yoo—Modified and Rosner, generate the minimal percentage deviation for the two morphological quantities, cell diameter and local density. Figure 9a illustrates that the Han and Yoo—Modified model in the context of a uniform prism mesh produces a percent deviation in cell diameter of 4.99%. The combination of the Han and Yoo—Modified model with a BLM configuration yields a percentage deviation of 5.09%. When considering local density, similar tendencies can be found. Here, the model according to Han and Yoo—Modified once again performed with the highest accuracy. The mean percentage deviation amounts to 14.56% (prismatic mesh) and 2.82% (BLM), respectively.

Likewise, the Rosner model performs at a relatively small error with respect to the average cell diameter. Thus, using a prism mesh, a percentage error of 9.40% is observed, and using a BLM configuration, an error of 11.11% is realized. For the local density, the error is more reduced, decreasing to 4.64% (BLM) and 17.36% (prism mesh). The models according to Han and Yoo, Payvar, and Shafi show a percentage deviation of at least 24.3% for the mean cell diameter. For the local density, on the other hand, a percentage deviation of at least 53.13% can be concluded for the models just mentioned.

**Figure 10:**
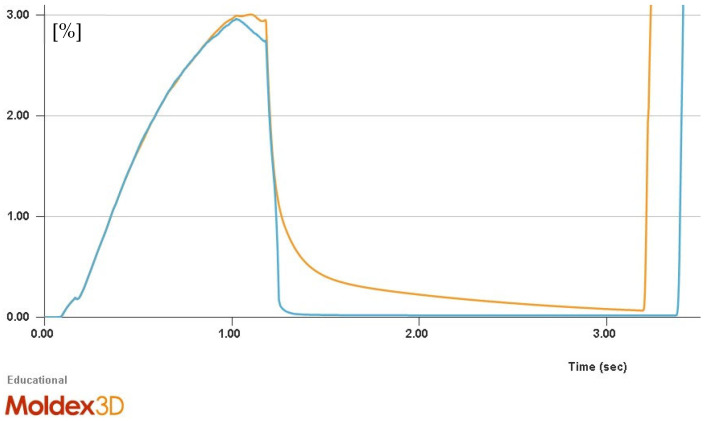
Dynamics (gas volume fraction) of bubble growth and shrinkage (blue = Han and Yoo, orange = Han and Yoo—Modified).

In past publications it has been discussed that the model according to Payvar does not consider variables such as surface tension and viscous effects, which can account for the inaccurate simulation [38]. If the algebraic sign of the average deviation of the cell diameter is considered, it can be noticed for the Payvar and Shafi bubble growth models that the model according to Payvar exhibits a larger positive deviation of the cell diameter and accordingly simulates bubbles that are too large. Shafi, on the other hand, displays a smaller positive deviation of the cell diameter. Hence, the average diameter of a bubble using the Payvar bubble growth model amounts to +41.74%. Regarding the Shafi model, the error amounts to +25.22%. Both values are given for the simulation with a prism mesh configuration (shown in Figure 9a). This phenomenon has already been theoretically described in this article and is also discussed and confirmed in other studies [13,15,23]. Still, the standard Han and Yoo model does not yield a higher significant simulation accuracy in this case. Although the standard Han and Yoo model specifies a viscoelastic polymer melt, this fact alone does not lead to a significant improvement of the simulation accuracy. In similar studies, a comparable conclusion was reached. Accordingly, the effect of viscoelasticity only appears to be important in the early stages of bubble growth and can be neglected in the later stages of growth [39]. Further, literature already pointed out that in alternative scenarios with different polymer and blowing agent combinations (such as PP/CO_2_) alternative bubble growth models other than Han and Yoo can prove to be advantageous [13]. Based on these findings, it can be postulated that irrespective of the mesh type, the bubble growth models according to Han and Yoo—Modified and Rosner show the highest simulation accuracy of foamed microcellular polycarbonates in the context of the Pull and Foam process. Since the specific models were designed with the condition of high-pressure levels, these theoretical models confirm the results that have been observed. To assess these findings, the dynamics of the bubble growth were analysed in the simulation. Figure 10 illustrates, thereby, the total gas volume fraction for the bubble growth models Han and Yoo and Han and Yoo—Modified.

Since we find a holding pressure of 60 MPa and thus a high pressure in the cavity during the Pull and Foam process, a regressive shrinking of the bubbles is to be expected after the initial nucleation of the bubbles, since a pressure build-up can be observed after the filling of the cavity. Figure 10 indicates that in the simulation the Han and Yoo—Modified model produces a more realistic shrinkage of the bubbles. By comparison with the literature, this assumption is confirmed, as the dynamics of the bubble growth follow a similar trend when considering the dynamics of cell size growth and shrinkage [10,11]. The Rosner model operates in an analogous pattern.

**Figure 11:**
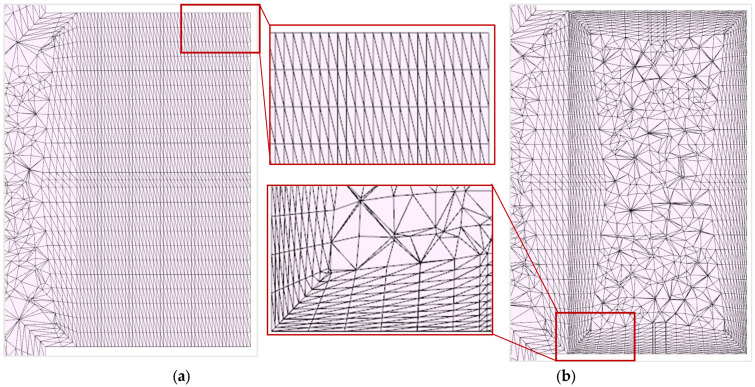
(**a**) Uniform prism mesh; (**b**) Hybrid BLM configuration with ten layers consisting of triangular prismatic elements at the boundary wall and tetrahedral elements at the center region.

From Figure 11 it can be seen that a BLM configuration with just ten outer prism mesh layers (Figure 11b)—irrespective of the bubble growth model—resulted in a higher simulation fidelity, although the uniform and pure prism or pentahedral mesh (Figure 11a) holds a higher average resolution. Since in principle, triangular prismatic as well as hexahedral meshes represent a higher mesh quality, the local mesh resolution and therefore element density respectively proves to be decisive in this case. Comparable investigations and deductions have been made in the research of fluid dynamics. According to these studies, different flow conditions require different mesh resolutions. However, these flow conditions are usually not known beforehand [19]. Process observations of various parameters, such as temperature and pressure curves, show that the strongest gradients are found normal to and near the boundary wall. At the center of a cross-section, however, the local changes for the above-mentioned parameters are relatively insignificant. Therefore, it can be concluded that for the Pull and Foam process simulation, the regions especially with the strongest gradients require a sufficiently high (above-average) resolution of the mesh configuration. Thus, the number of elements in the direction from the edge to the center region is decisive. Prisms should therefore be modelled in sufficient quantity at the boundary regions. Furthermore, based on these findings, it can be stated that the theoretically inferior tetrahedral elements in the center of the BLM do not result in a reduction of the simulation quality, although the experimental as well as the simulative measurement volume element (as shown in Figure 4) can be found in this region. In this case, at the boundary region, the uniform pure prism mesh features an element density of 28 prismatic elements per mm^3^. In contrast, the hybrid BLM provides a prismatic element density of 82 elements per mm^3^. In addition, it has been explained in further literature that triangular prismatic elements generally have a higher number of neighbours than tetrahedral elements, which feature four neighbours, which leads to a better approximation of gradients and thus to a higher simulation accuracy [18]. Therefore, a high mesh resolution, respectively a high element density, is to be preferred in locations with strong gradients in order to increase the number of elements and thus the number of neighbours.

**Figure 12:**
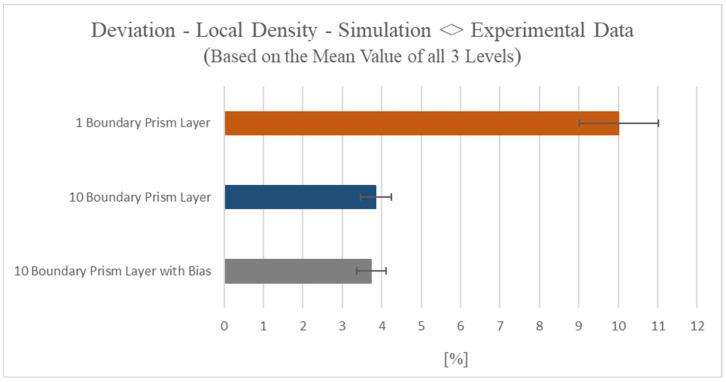
Illustration of the percentual deviation (error) of the local density between experimental and simulation data depending on the local mesh resolution.

In order to investigate this issue in further detail, complementary investigations were conducted with respect to the variation of the local mesh resolution of the prism layers of the hybrid BLM. The respective bubble growth model used corresponded to the model according to Han and Yoo—Modified. Hence, analogously to Figure 7b, for all 30 datapoints each for the top level, center level, and bottom level, the local density was simulated and evaluated with these different BLM configurations. Figure 12 shows the percentage deviation (error) of all three mesh configurations. The structural characterization of the experimental samples showed an average density of 0.841 g/cm^3^ (standard deviation of 0.12, *n* = 30) and this value serves as reference for the simulated densities and as calculation basis for the resulting error.

For the first case, the boundary region of the hybrid BLM configuration was meshed in such a manner that the boundary region consisted of only one prism layer. Since in this case the simulated average local density amounts to 0.934 g/cm^3^ (standard deviation of 0.169), this mesh configuration yields an error of 10.2%. In the instance of ten prism layers each with identical thickness, an average error of 3.85% is generated as the average local density is simulated with 0.874 g/cm^3^ (standard deviation of 0.121). If the boundary layer is meshed with ten prism layers with a variation regarding the element thickness, the individual triangular prismatic elements decrease in thickness the closer the elements are situated to the boundary region (bias). Here, only a minor increase of the simulation accuracy can be observed since the error amounts to 3.71%. The corresponding calculated average local density amounts to 0.873 g/cm^3^ (standard deviation of 0.125). From these results, it is thus observed how the boundary mesh resolution, respectively the quantity of prismatic elements and therefore prism layers, impacts the simulation accuracy.

## 4. Conclusions

In this article, the special foam injection molding process Pull and Foam, which is based on a precision mold opening technology and on a MuCell^®^ enhanced Arburg Allrounder 470S injection molding machine, was simulated using the filling simulation software Moldex 3D. Characteristic morphological parameters, such as cell diameter and local density were compared. For the experimental characterization of the structure, digital light microscopy and X-ray microtomography images were generated, which were digitally and quantitatively analyzed using the software Avizo and Photoshop. In order to vary different simulation settings, different mesh type configurations were parameterized and examined. On the one hand, a uniform mesh configuration of triangular prismatic elements was defined for the expansion zone of the foamed sample. On the other hand, a hybrid boundary layer mesh (BLM) was defined, which contains triangular prismatic elements at the boundary region and unstructured tetrahedral elements at the center. It can be shown that a uniform prism mesh, which provides a general high mesh resolution, does not result in a higher accuracy simulation for the Pull and Foam process simulation. The BLM configuration with its locally above-average mesh resolution at the boundary wall can yield a more accurate calculation of quantities such as cell diameter and local density. This can be explained by the fact that typical Pull and Foam process parameters and morphological variables (local density, cell diameter, temperature, pressure profile) and their trends have the strongest variation, respectively the strongest gradient at the boundary region. Since these gradients, which extend normal from the boundary wall, show an increasingly flattening trend up to the center, priority must be given to high-resolution meshed boundary regions for this case. The meshing, respectively the meshing resolution at the center, is therefore to be regarded as secondary. Furthermore, in this study, different bubble growth models known as Han and Yoo, Han and Yoo—Modified, Shafi, Payvar as well as Rosner were varied to evaluate their simulation accuracy in the simulation. For the simulation of the Pull and Foam process, the Han and Yoo—Modified and Rosner model offered the most valid simulation of the nucleation and the bubble growth dynamics. Since these equations describe a more realistic cell growth and shrinkage, especially at high pressure levels, it is beneficial to apply them to the Pull and Foam process simulation.

## Figures and Tables

**Table 1:** Relevant material properties of polycarbonate (Makrolon^®^ 2405).

Melt Volume Rate (MVR)	Density (ρ)
19 cm^3^/10 min (300 °C—1.2 kg)	1.20 g/cm^3^

## Data Availability

The data presented in this study are available on request from the corresponding author. The data are not publicly available due to the large size.
